# A Cyclic GMP Signalling Module That Regulates Gliding Motility in a Malaria Parasite

**DOI:** 10.1371/journal.ppat.1000599

**Published:** 2009-09-25

**Authors:** Robert W. Moon, Cathy J. Taylor, Claudia Bex, Rebecca Schepers, David Goulding, Chris J. Janse, Andrew P. Waters, David A. Baker, Oliver Billker

**Affiliations:** 1 Wellcome Trust Sanger Institute, Cambridge Hinxton, United Kingdom; 2 Imperial College London, Department of Cell and Molecular Biology, London, United Kingdom; 3 Department of Infectious and Tropical Diseases, London School of Hygiene & Tropical Medicine, London, United Kingdom; 4 Department of Parasitology, Centre of Infectious Diseases, Leiden University Medical Centre, Leiden, The Netherlands; 5 Wellcome Trust Centre of Molecular Parasitology and Division of Infection and Immunity, University of Glasgow, Glasgow, United Kingdom; Washington University School of Medicine, United States of America

## Abstract

The ookinete is a motile stage in the malaria life cycle which forms in the mosquito blood meal from the zygote. Ookinetes use an acto-myosin motor to glide towards and penetrate the midgut wall to establish infection in the vector. The regulation of gliding motility is poorly understood. Through genetic interaction studies we here describe a signalling module that identifies guanosine 3′, 5′-cyclic monophosphate (cGMP) as an important second messenger regulating ookinete differentiation and motility. In ookinetes lacking the cyclic nucleotide degrading phosphodiesterase δ (PDEδ), unregulated signalling through cGMP results in rounding up of the normally banana-shaped cells. This phenotype is suppressed in a double mutant additionally lacking guanylyl cyclase β (GCβ), showing that in ookinetes GCβ is an important source for cGMP, and that PDEδ is the relevant cGMP degrading enzyme. Inhibition of the cGMP-dependent protein kinase, PKG, blocks gliding, whereas enhanced signalling through cGMP restores normal gliding speed in a mutant lacking calcium dependent protein kinase 3, suggesting at least a partial overlap between calcium and cGMP dependent pathways. These data demonstrate an important function for signalling through cGMP, and most likely PKG, in dynamically regulating ookinete gliding during the transmission of malaria to the mosquito.

## Author Summary

Malaria parasites are single celled organisms, which must alternate between vertebrate and mosquito hosts to survive and spread. In both hosts, certain parasite stages can glide through tissues and invade cells. Many components of the molecular motor that powers gliding and invasion are known and we have a good idea how these may interact to generate force. It is less well understood how the motor is assembled and how its component parts are regulated to switch it on and off. We have begun to address these questions in the ookinete, a parasite stage, which forms in the blood meal of a mosquito and relies on gliding to penetrate the gut wall. Using a malaria parasite of rodents, we have examined the effect of deleting candidate genes involved in controlling levels of the intracellular signalling molecule cyclic guanosine monophosphate (cGMP). We show that the right balance between cGMP production and degradation is important for ookinetes to glide, while also maintaining their typical cell shape. Overall levels of cGMP are not much affected in the mutants, though, and we therefore believe the messenger exerts its effect either locally within the cell or only while the parasite is gliding.

## Introduction

Malaria parasites belong to the subphylum apicomplexa, which comprises a large diversity of often intracellular parasites, including important causative agents of disease in humans and animals. Apicomplexa use a unique kind of substrate dependent gliding motility as a key virulence strategy [1]–[3]. Gliding enables some parasite stages to actively seek out and penetrate host tissues and also powers host cell invasion. Once parasites have matured within and then lysed an infected host cell, gliding can accompany parasite egress and mediate dispersal [4]. Malaria parasites rely on gliding to colonise both their vertebrate host and their mosquito vector. Sporozoites delivered into the skin with the saliva of an infectious mosquito actively glide through the dermis, penetrate the endothelial wall of blood vessels [5]. Once in the liver, sporozoites pass through cells of the liver before invading a hepatocytes by forming a parasitophorous vacuole [6]. The second malaria zoite capable of gliding is the ookinete, which forms in the mosquito blood meal and is essential for parasite transmission back to the vector. Transmission requires the ingestion of red blood cells infected with specialised sexual precursor stages, the gametocytes, into the blood meal of a vector, where a mosquito factor triggers the rapid differentiation into gametes [7]. Fertilisation is followed by meiosis, and within 24 h the zygotes transform into ookinetes, which move actively through the blood meal, penetrate the mosquito-derived peritrophic matrix that encloses the blood bolus, and cross the epithelial monolayer of the mosquito midgut, before lodging themselves between the midgut basal lamina and the epithelium [8]. Here ookinetes transform into oocysts, which eventually release sporozoites and invade the salivary glands.

Apicomplexan zoites all share a highly polarized cellular organisation that reflects their similar colonisation strategies. Conserved features include the apical complex composed of secretory organelles and a polar ring that functions as an apical organising centre, from which varying numbers of microtubules emanate that run along the length of the cell. These are connected to the inner layer of a three-membrane pellicle composed of the plasmalemma and the underlying inner membrane complex (IMC). Gliding and invasion are powered by an actomyosin-based molecular motor contained within the narrow cytosolic space between the parasite's plasma membrane and the IMC [9]. This motor generates force by translocating stage and species specific transmembrane adhesins of the TRAP/MIC2 family from their apical point of secretion towards the posterior pole of the cell, thereby pushing the parasite forward. The molecular composition of the motor has been studied mostly in *Toxoplasma gondii* tachyzoites and *Plasmodium* sporozoites but is thought to be conserved in all gliding stages, as well as in erythrocyte invasion by *Plasmodium* merozoites [10],[11]. Components of the motor include the glycolytic enzyme aldolase, which links the conserved cytoplasmic C-terminus of the TRAP/MIC2 family adhesins to actin filaments [12], and a class XIV myosin, MyoA, with its light chain [13], referred to as MTIP in *Plasmodium* [14]. The motor complex interacts with two novel gliding associated proteins, GAP45 and GAP50, the latter of which anchors it to the outer membrane of the IMC [15].

Ookinete gliding requires mobilisation of calcium from internal stores [16], as does microneme secretion, motility and invasion in other apicomplexan zoites [17],[18]. Important calcium effector proteins of apicomplexa include a family of calcium dependent protein kinases (CDPKs), which the parasites share with ciliates and plants [19]. In *P. berghei*, a malaria parasite of rodents, a member of this family, *Pb*CDPK3, is required for efficient gliding of ookinetes [16],[20]. In *T. gondii* tachyzoites pharmacological evidence had previously indicated a role for a different CDPK in motility and attachment [21],[22]. The molecular substrates of *Pb*CDPK3 in ookinetes remain unknown, but in *P. falciparum* yet another member of the family, *Pf*CDPK1, localises to the periphery of the merozoite, phosphorylates GAP45 and MTIP *in vitro* [23], and is the target of a selective inhibitor that blocks merozoite maturation or invasion [24], suggesting that phosphorylation of motor components may be one way in which a calcium/CDPK pathway can regulate gliding. In support of this hypothesis, phosphorylation of *T. gondii* GAP45 is likely required for assembly of the motor complex [25].

Emerging evidence suggests another layer of regulation is provided by cyclic guanosine 3′, 5′-cyclic monophosphate (cGMP) dependent signal transduction pathways. In *T. gondii* tachyzoites selective inhibition of the parasite's cGMP dependent protein kinase (PKG) by the trisubstituted pyrrole 4-[2-(4-fluorophenyl)-5-(1-methylpiperidine-4-yl)-1H-pyrrol-3-yl] pyridine, compound 1 (Cmpd 1), blocks motility and invasion [26]. In *P. berghei* disruption of a gene encoding a cGMP producing enzyme, guanylyl cyclase β (GCβ), resulted in a motility defect in ookinetes [27]. Previous work with *P. falciparum* GCβ has shown that recombinant cyclase domains of this cyclase produces cGMP and strictly prefers GTP over ATP as substrate [28]. However, a role for cGMP signalling in ookinete motility is not the only possible explanation for the phenotype of the *gcβ* mutant since GCβ also contains a large ATPase-like domain of unknown function. Signalling though cGMP is not limited to zoite stages, but also required for *Plasmodium* sexual development. Gametocyte activation in *P. falciparum* requires PKG [29], and deletion of a cGMP degrading phosphodiesterases, PDEδ, results in accumulation of cGMP in late stage gametocytes, which is accompanied by the failure of these cells to become fully responsive to triggers of activation [30].

Recognising that *P. berghei* ookinetes offer a genetically very accessible system to dissect signal transduction pathways, we have combined pharmacological and genetic interaction studies to identify cGMP signalling genes involved in motility. We demonstrate that in *P. berghei* at least three gene products interact in a cGMP signalling module that is critically required for ookinete morphology and gliding. We present evidence supporting a model in which GCβ and PDEδ, by producing and hydrolysing cGMP, regulate levels of cGMP, which is required for ookinete gliding. We also present more tentative evidence suggesting PKG is the effector of cGMP in the ookinete.

## Results

### Cyclic nucleotide signalling genes of *P. berghei*

To identify candidate genes for cyclic nucleotide signalling in *P. berghei* we searched the malaria genome database, PlasmoDB [31]. We found evidence for four putative nucleotide cyclases, four class I phosphodiesterases, one gene for the effector kinase PKG and two genes encoding the catalytic and regulatory subunits of PKA, respectively (Table S1). Each of these has an ortholog in the human malaria parasite, *P. falciparum*, suggesting cyclic nucleotide signalling is in principle conserved in both species. Our search also found a novel putative cyclic nucleotide effector gene conserved in apicomplexa and predicted to encode a large (∼420 kDa) protein with signatures of 5 cyclic nucleotide binding domains, only the most C-terminal of which contains all residues for nucleotide binding, including a threonine residue diagnostic of specificity for cGMP [32]. Conserved effector domains appear to be lacking from this protein, leaving its function uncertain. We did not find candidates for cyclic nucleotide gated ion channels in *Plasmodium*.

### Ookinete gliding requires *gcβ*

**Figure 1:**
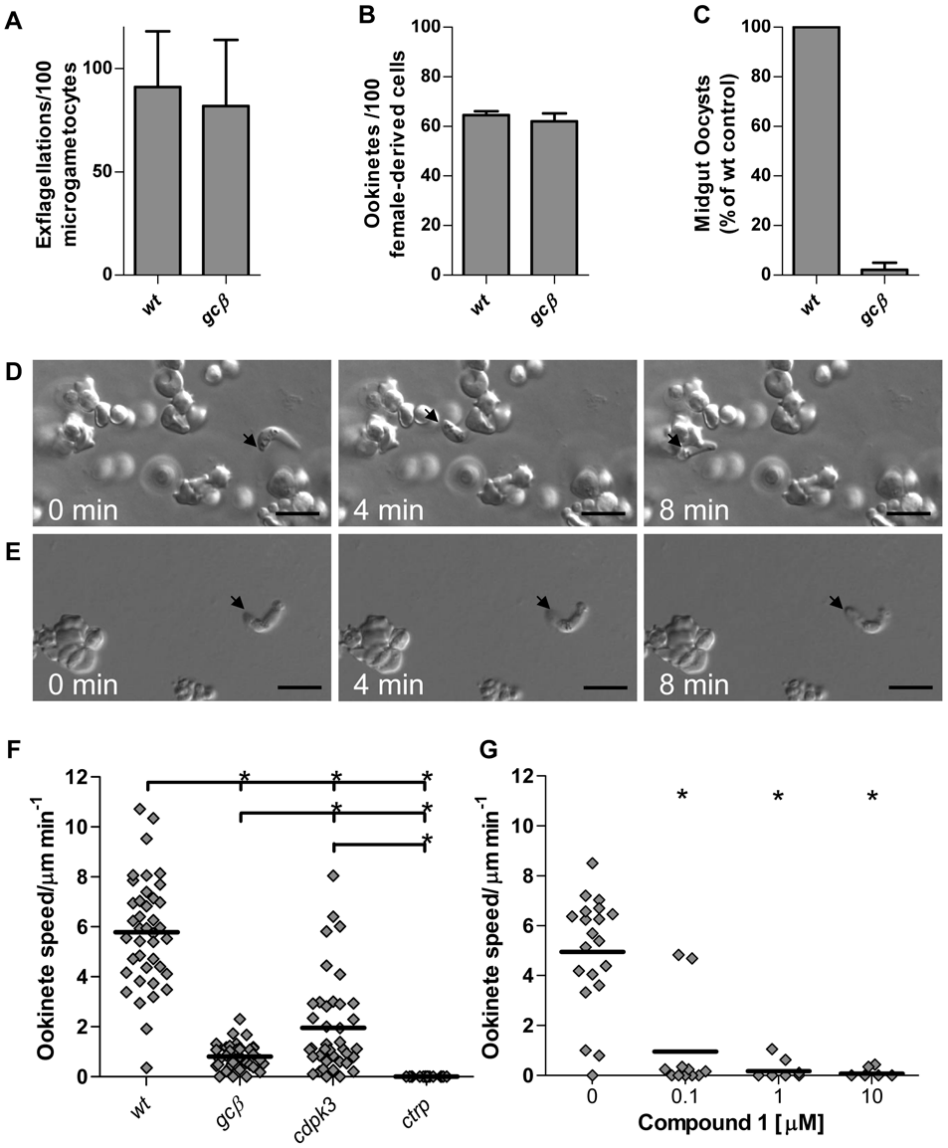
GCβ is essential for ookinete motility and parasite transmission to the mosquito. (A) Male gamete formation (exflagellation), (B) macrogamete-to-mature ookinete conversion rate (determined as % of “banana”-shaped cells stained with female-derived cell specific antibody) and (C) average oocysts numbers on midguts dissected 10 days post infection were compared. Data were from 3 independent experiments and error bars show standard deviations. Different gliding speeds of wild type (D) and *gcβ* (E) ookinetes in Matrigel™ is illustrated with representative frames from time lapse videos (Videos S1 and S2). The black arrow marks the apical end of the ookinete and scale bar denotes 10 µm. (F) Speed of individual ookinetes (diamonds) from 24 h cultures, measured over a 10 min period in 3 mutants compared to wild type. The thick black lines denote mean. (G) Impact of Cmpd 1 on gliding of wild type ookinetes. * = significantly different, Student's T-test, p≤0.01.

Two of the four nucleotide cyclases, *Pb*GCα and *Pb*GCβ, are predicted to generate cGMP. Both possess the unusual domain organisation first described for their *P. falciparum* orthologues [28], which contain N-terminally 10 predicted transmembrane domains that form a P-type ATPase-like domain, and C-terminally a cyclase domain composed of two catalytic domains, each preceded by 6 transmembrane regions. Real time PCR found *gcα* to be the dominant guanylyl cyclase in mixed asexual erythrocytic stages, whereas enriched gametocytes additionally transcribed *gcβ* (Fig. S1A). This is in agreement with data from genome-wide transcriptional profiling in *P. falciparum* [33],[34]. Repeated attempts to disrupt *P. berghei gcα* by inserting an antimalarial drug resistance marker failed (data not shown), suggesting this cyclase may have an essential function in asexual erythrocytic stages. In contrast, parasites in which *gcβ* was disrupted were readily obtained by us (Fig. S2A–C) and in an independent study [27]. Consistent with the earlier work, our *gcβ* mutant produced normal numbers of micro- and macrogametocytes, which could be triggered to undergo exflagellation and fertilize *in vitro* (Fig. 1A). Normal numbers of *gcβ* zygotes formed in culture and differentiated into ookinetes (Fig. 1B). However, in mosquito transmission experiments, *gcβ* parasites were far less successful than wild type. When we examined midguts 10 days after *A. stephensi* mosquitoes had fed on infected mice we observed only 2.2% of wild type oocysts (Fig. 1C), indicating a phenotype at or after the ookinete stage. Our analysis concurs entirely with the characterisation of a similar mutant by Hirai et al. [27], who concluded *gcβ* is required at the stage of ookinete invasion of the mosquito midgut epithelium, and who identified a defect in gliding motility in the *gcβ* mutant.

In-depth phenotyping of gliding mutants required an assay, in which sufficient numbers of moving ookinetes could be recorded by time-lapse video microscopy and motility parameters quantified. We developed such an assay based on our observation that embedding cultured ookinetes in a dilute gel of mouse extracellular matrix components (Matrigel™) elicits productive gliding in the vast majority of ookinetes, while preventing passive cell movements. It was necessary to reduce the density of the gel by dilution with medium, such that wild type ookinetes followed a characteristic helical gliding path and moved at an average speed of 5.8 µm/min (Fig. 1D and Video S1). These parameters matched closely those determined for gliding of GFP-expressing ookinetes within the mosquito blood meal [8]. To test this assay we first examined an existing motility mutant, in which the gene for circumsporozoite- and TRAP-related protein (*ctrp*) had been disrupted [35]. As expected, *ctrp* ookinetes displayed a complete loss of forward motility (Fig. 1F), although occasional bending was still observed (not shown). This is consistent with the essential role CTRP is thought to play as the transmembrane adhesin that links the extracellular substrate to the submembrane actomyosin motor of the ookinete. Ookinetes lacking the CDPK3 kinase [20] also had a significantly reduced gliding speed that was intermediate between wild type and *ctrp*, confirming our previous observations. *gcβ* ookinetes had a more severely reduced motility, with only rare bouts of slow gliding (Fig. 1E and Video S2) resulting in a stronger reduction in average speed when compared to *cdpk3* (Fig. 1F).

### Gliding is sensitive to an inhibitor of PKG

The mechanism through which *gcβ* affects gliding could depend on the confirmed ability of GCβ's guanylyl cyclase domain to generate the secondary messenger cGMP [28], or the motility defect might be due to a role of the uncharacterised P-type ATPase-like domain. Only the former would be expected to activate cGMP effector pathways, which could then modulate gliding either through PKG, or through the novel putative cyclic nucleotide binding protein. In support of a role for cGMP and PKG, we found that a selective inhibitor of apicomplexan PKG, the ATP analog cmpd 1 [36], potently blocked gliding of wild type ookinetes with a half-maximal effect below 100 nM (Fig. 1G). The *pkg* gene proved refractory to targeted disruption suggesting an essential role in the asexual erythrocytic stages and we were therefore unable to confirm its role in gliding directly. The *pkg* locus was, however, accessible to genetic modification, allowing us to generate a genomic 3′ fusion with *gfp* (Fig. S3A). A PKG-GFP fusion protein of the expected size was expressed from the endogenous *pkg* promoter at similar levels in mixed erythrocytic stages, gametocytes and ookinetes (Fig. S3B, C), consistent with a role for PKG in all these life cycle stages. The diffuse cytosolic distribution of the fusion protein provided no further insights into its specific functions (Fig. S3C).

### Ookinete morphology relies on *pdeδ*

**Figure 2:**
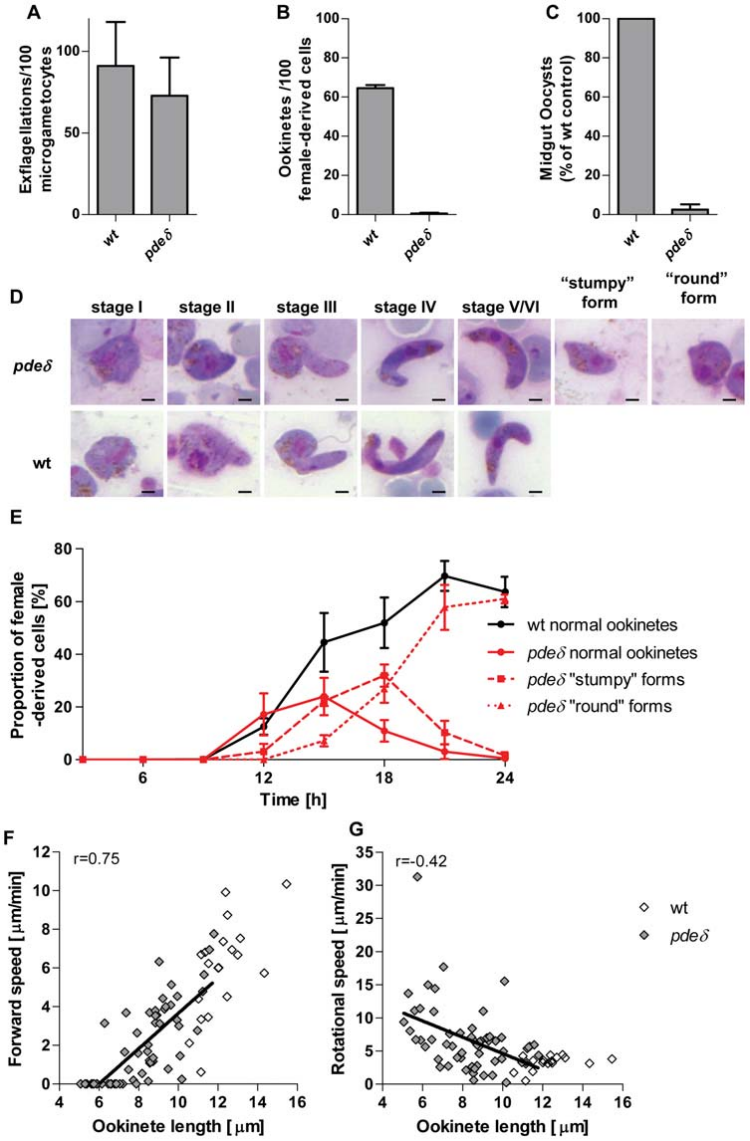
PDEδ is essential for normal ookinete morphology and parasite transmission to the mosquito. (A) Male gamete formation (exflagellation), (B) macrogamete-to-mature ookinete conversion rate (determined as % of “banana”-shaped cells stained with female-derived cell specific antibody) and (C) average oocysts numbers on midguts dissected 10 days post infection were compared. Data were from 3 independent experiments and error bars show standard deviations. (D) Giemsa-stained blood films illustrating normal stages of ookinete differentiation and aberrant forms additionally present in *pdeδ* ookinete cultures. Scale bars indicate 2 µm. (E) Ookinete stages as a percentage of the total number of female derived cells on Giemsa-stained blood films. Slides were prepared from *in vitro* cultures at the times indicated. Error bars represent standard deviations in three biological replicates. (F) Forward motility and (G) rotation speed of *pdeδ* and wild type ookinetes as a function of cell length from 15–22 hour *in vitro* cultures were determined using the Matrigel™ motility assay. Forward speed was averaged over 10 minutes. The rotational component of ookinete motility was determined as (number of turns×circumference at widest point) and averaged over 10 min. Best fit and correlation coefficient r are given for *pdeδ* ookinetes only. Examples of motility of *pdeδ* ookinetes with normal, stumpy and round morphology are shown in Videos S3, S4 and S5.

Since gliding appeared to involve cGMP, we sought to establish which of the four cyclic nucleotide degrading enzymes is involved in negatively regulating cGMP during sexual development. Transcriptional profiling in *P. falciparum* asexual and sexual erythrocytic stages [33],[34] had shown PfPDEγ and PfPDEδ to be up regulated in gametocytes, suggesting possible functions in sexual development. Expression analysis of all four cyclic nucleotide phosphodiesterases in mixed asexual erythrocytic stages and gametocytes by real time PCR confirmed that PDEγ and PDEδ were both highly expressed in *P. berghei* sexual stages (Fig. S1B). We first generated a *pdeγ* deletion mutant, which was viable and had no discernible phenotype up to and including the oocyst stage, suggesting this phosphodiesterase was not essential during sexual development (not shown). We next targeted the *pdeδ* gene (Fig. S2D) and confirmed the genotype of a mutant clone by PCR (Fig. S2E) and Southern blot analysis (Fig. S2F). The asexual growth rate of blood stages, their ability to produce gametocytes, and *in vitro* gametocyte activation of the *pdeδ* mutant (Fig. 2A) were as in wild type. However, when cultured for 24 h *in vitro*, hardly any typically shaped ookinetes were present (Fig. 2B). In transmission experiments, oocyst numbers on the mosquito midgut epithelium were reduced by >94% (Fig. 2C). Upon closer inspection we found ookinete cultures were dominated by stumpy or round cells (Fig. 2D) each with a single small protrusion, which we tentatively identified as remnants of an apical complex. We therefore speculated that *pdeδ* zygotes were able to undergo some kind of cellular differentiation. Time course experiments confirmed that during the first 12 h of *in vitro* culture, *pdeδ* zygotes differentiated into morphologically advanced ookinetes (stages IV–VI as described by [37]) in similar numbers as wild type (Fig. 2E). In wild type cultures mature forms continued to accumulate, reaching 60% of all macrogamete-derived parasites by 24 h, the remainder presumably being unfertilised macrogametes. In contrast, all morphologically mature ookinetes in early *pdeδ* cultures were replaced successively by stumpy and round forms (Fig. 2E).

### A *pdeδ* mutant links ookinete shape to productive gliding

Aberrant differentiation of *pdeδ* ookinetes was not accompanied by parasite death, as judged initially by exclusion of the membrane impermeable SYTOX® green nucleic acid stain (data not shown), and by the fact that total parasite numbers remained similar to wild type throughout the 24 h culture period. In fact, when we bypassed the physical barrier posed by the midgut epithelium by injecting dedifferentiated *pdeδ* ookinetes directly into the mosquito haemocoel, salivary gland infections were boosted to levels observed with wild type ookinetes delivered by the same route (Table 1). Wild type and *pdeδ* injected mosquitoes could transmit the parasites back to mice. In contrast, injection of *gcβ* ookinetes did not restore salivary gland infection (Table 1), raising the possibility of an additional function for GCβ subsequent to the ookinete stage.

**Table 1:** Sporozoite numbers and infectivity in ookinete injected mosquitoes.

	Clone	Average number of salivary gland sporozoites (*)	Mice infected/mice fed (Ψ)
Exp.1	wild type	10828 (17)	2/2 (25)
	*gcβ*	0 (28)	0/2 (25)
	*pdeδ*	17513 (23)	2/2 (25)
Exp. 2	wild type	13527 (13)	2/2 (15)
	*gcβ*	0 (16)	0/2 (20)
	*pdeδ*	5010 (17)	2/2 (10)
Exp. 3	wild type	3929 (16)	1/1(40)
	*gcβ*	0 (14)	0/1(40)
	*pdeδ*	5522 (20)	1/1(17)

20–21 days after injection with mutant and wild type ookinetes, salivary glands were dissected and sporozoites were quantified, or mosquitoes were allowed to feed on naïve mice. * Number of mosquitoes dissected. Ψ Number of mosquitoes fed.

Haemocoel injections suggested that the transmission phenotype of *pdeδ* ookinetes does not result from a lack of cellular viability *per se*, but from the parasite's inability to cross the midgut epithelium. We therefore examined the relationship between cell morphology and motility. In wild type ookinetes the subpellicular microtubules, which are thought to dictate the orientation of the molecular motor, run at a slight angle with the longitudinal axis of the cell, and as a result the motor generates a strong forward and a weaker rotational force [3],[38]. Together with the crescent shape of the cell, these combined forces give rise to the helical path that is typical of ookinete gliding in a three dimensional matrix (see for instance Video S1). Measuring separately forward and rotational motility components in a spectrum of normal to aberrant *pdeδ* ookinetes and in wild type, we observed a positive correlation (r = 0.75) between ookinete length and forward motility (Fig. 2F). *Pdeδ* ookinetes with normal morphology moved at speeds comparable to wild type, while round *pdeδ* ookinetes produced little or no forward motility. Conversely, rotational speed increased with the severity of the morphological defect, such that round *pdeδ* ookinetes were seen to rotate rapidly on the spot (Fig. 2D and Video S5). Disruption of *pdeδ* thus did not appear to affect motor activity, but as a result of changed cellular morphology it reduced forward gliding in a way that could explain the marked reduction in natural mosquito transmission.

**Figure 3:**
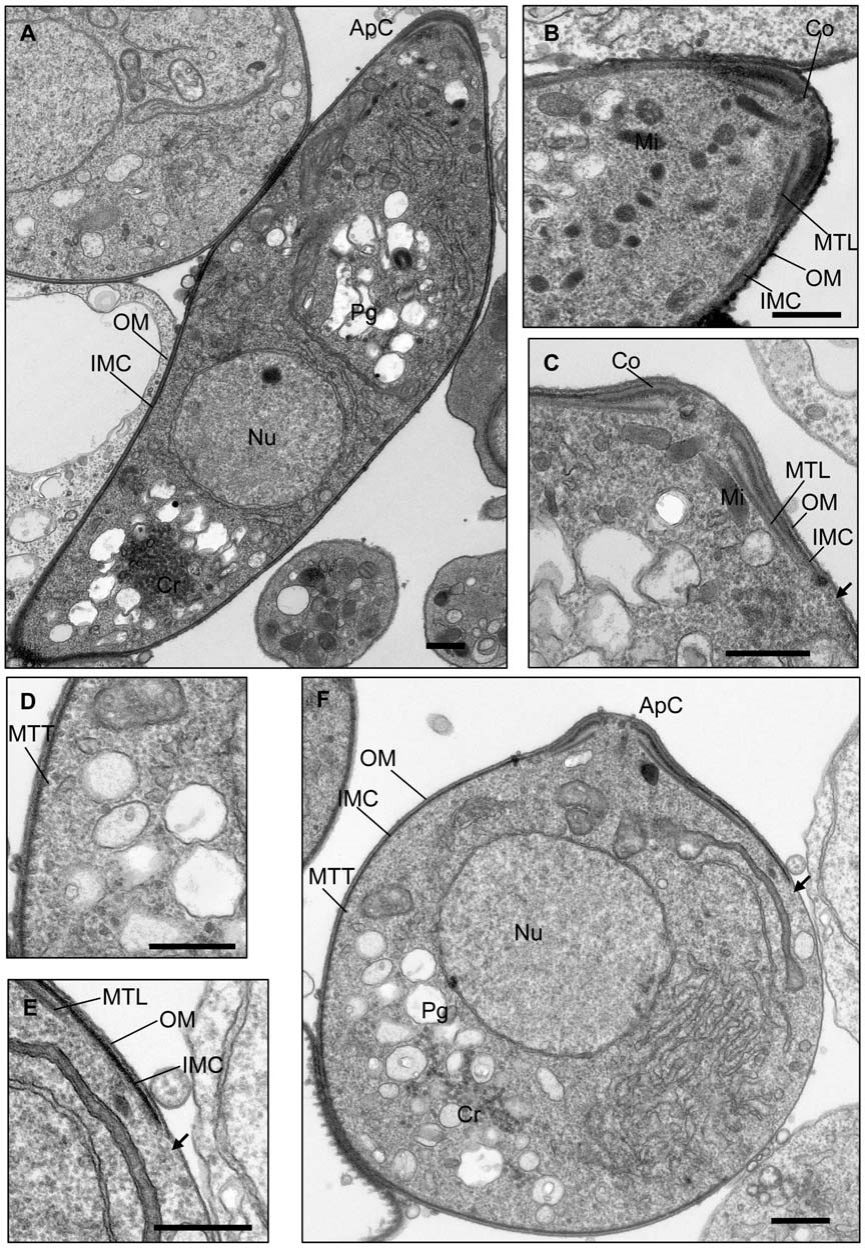
Transmission electron micrographs of wild type (A, B) and *pdeδ* (C–F) ookinetes. (A) Sagittal section through wild type ookinete. (B). Detail of wild type apical complex. (C) Detail of *pdeδ* apical complex. (D) Detail of *pdeδ* subpellicular microtubules in transverse section (detail of F). (E) Detail of *pdeδ* IMC (detail of F). (F). Sagittal section of whole “round” form *pdeδ* ookinete. Arrows point to interruptions in the IMC (C, E and F). Scale bars denote 500 nm in all images. Abbreviations: ApC; Apical Complex, Co; Collar, Cr; Crystalloid, Pg; Pigment Vacuole, IMC; Inner Membrane Complex, Mi; Micronemes, MTL; Subpellicular Microtubules in Longitudinal Section, MTT; Subpellicular Microtubules in Traverse Section, Nu; Nucleus, OM; Outer Pellicle Membrane.

Consistent with this hypothesis, when we examined the ultrastructure of 24 h wild type (Fig. 3A, B) and dedifferentiated *pdeδ* ookinetes (Fig. 3C–F), we found the organisation of the mutant only mildly disrupted. The apical complex of *pdeδ* ookinetes appeared normal (Fig. 3B, C). Cytoskeletal structures composed of the polar rings and collar and micronemes, the secretory organelles usually found aggregated at the apical end of invasive stages, were present, as was the ookinete-specific crystalloid, an organelle of unknown function (Fig. 3A, F). In wild type ookinetes – as in all apicomplexan zoites – the apical cytoskeletal structures led on to the inner membrane complex (IMC), a double membrane beneath the plasmalemma, which in *Plasmodium* forms a continuous structure made from a large flattened vesicle with associated particles that ends near the posterior of the ookinete [39]. In contrast, longitudinal sections of *pdeδ* ookinetes invariably revealed a discontinuous IMC, with gaps which varied in size between sections, but on average accounted for 20% of the cell's circumference (Fig. 3E and F). Subpellicular microtubules, which in wild type run longitudinally in a slightly helical array beneath the IMC (Fig. 3B), were also seen in *pdeδ* ookinetes, except in regions where the IMC was interrupted (Fig. 3D, E). In many longitudinal sections of *pdeδ* ookinetes subpellicular microtubules were sectioned longitudinally where they emerged from the apical collar, but were cut transversely further away from the apical end, suggesting they ran at an almost right angle to the anterior-posterior axis of the cell (Fig. 3D–F). This provides a mechanistic explanation for the predominance of the rotational component in *pdeδ* ookinete motility. For comparison we also investigated *gcβ* ookinetes by transmission electron microscopy but found no ultrastructural abnormalities in this mutant (data not shown).

### Interactions between *pdeδ*, *gcβ* and PKG

**Figure 4:**
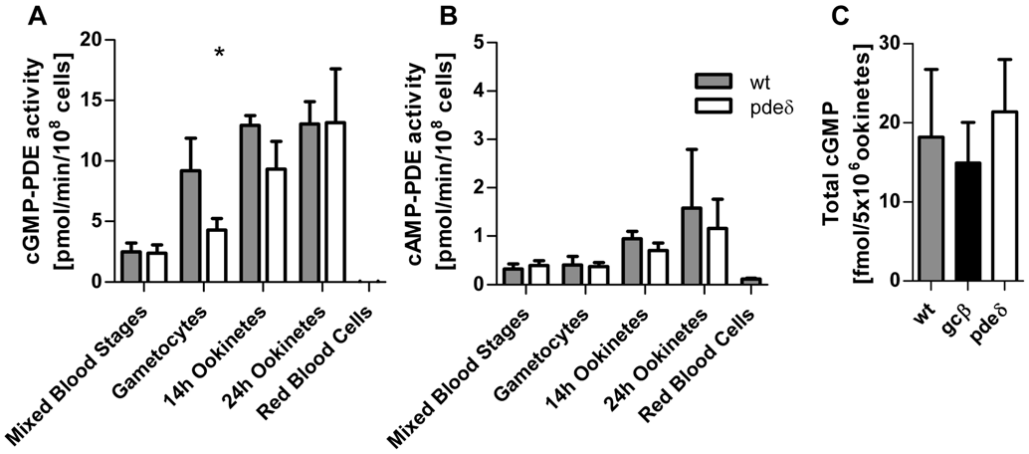
Biochemical analysis of *pdeδ* and *gcβ* mutants. cGMP-PDE (A) and cAMP-PDE (B) activity was measured in membrane fractions from various purified parasite stages in wild type and *pdeδ* parasites. (C) Total intracellular cGMP concentration was measured in 24 h paramagnetic bead-purified ookinetes. For all datasets the graphs show the mean of three independently purified samples in each case, with error bars indicating standard deviations.

**Figure 5:**
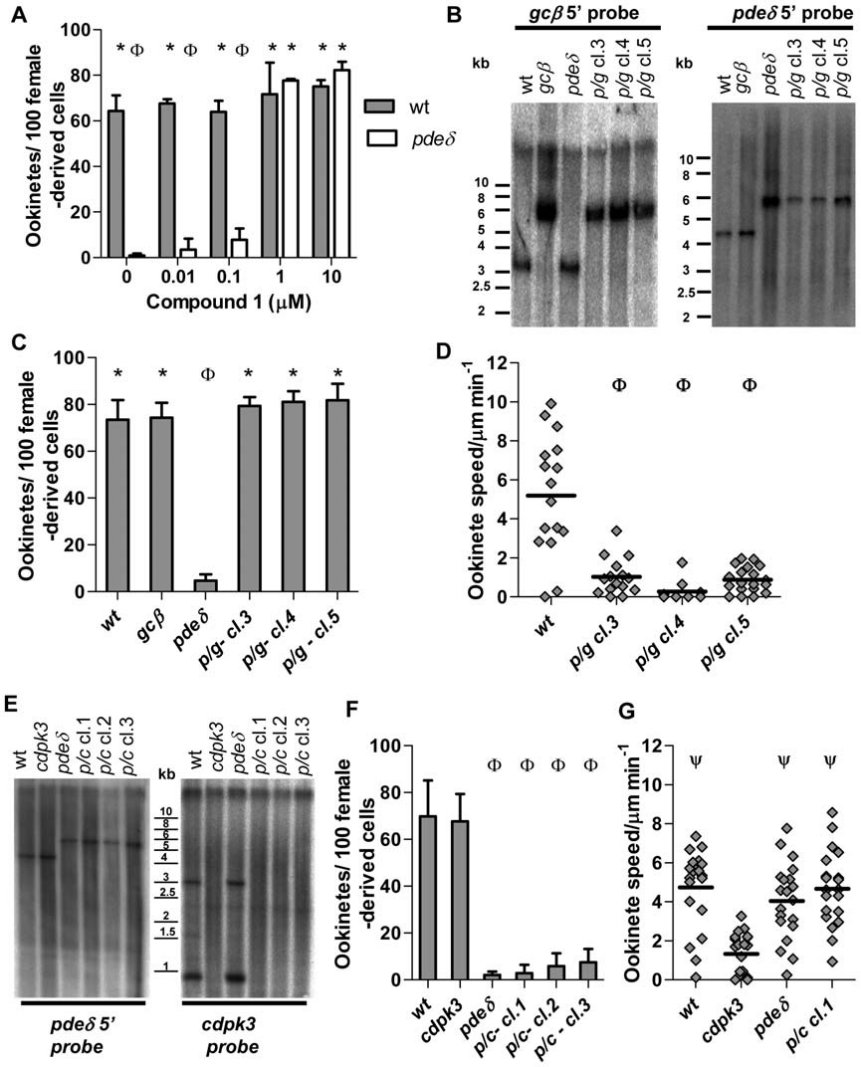
Suppression of the *pdeδ* phenotype by Cmpd 1 and epistatic interactions in *pdeδ gcβ* and *pdeδ cdpk3* double mutants. (A) Conversion rate to mature ookinetes was scored for 22 h cultures, to which Cmpd 1 or vehicle had been added from 3 hours of culture. (B) Southern blot analysis comparing genotypes of three *pdeδ gcβ* double knockout clones (*p/g* cl. 3–5) obtained from a cross to the parental mutants and wild type. Genomic DNA was digested and probed as for single mutants (Figs. 2 and 5). Effect of *pdeδ gcβ* double mutant (C), and pde*δ* cdpk3 double mutant (F) on conversion to morphologically mature ookinetes in 22-hour cultures. (D) Effect of *pdeδ gcβ* double mutant on average gliding speed in Matrigel™. (E) Three parasite clones obtained from the *pdeδ* and *cdpk3* genetic cross (*p/c*) were genotyped by Southern hybridization, along with wild type and parental clones. To examine disruption/deletion of the targeted locus genomic DNA was digested and probed as in Fig. 5 for *pdeδ*, or as described previously (Siden-Kiamos *et al.*, 2006) for *cdpk3*. In the left panel a 4.3 kb band is recognised by a 5' probe in *Eco*RI-digested DNA in wild type and appearance of a larger 5.8 kb fragment is diagnostic of *pdeδ* deletion. In the right panel a 3 kb and a 0.9 kb fragment recognised in *Eco*RI/*Hin*dIII digested DNA by a *cdpk3*-specific probe indicate an intact *cdpk3* locus only in wild type and *pdeδ*, and there absence indicates deletion of *cdpk3* in all other clones (see Figure S2 G). (G) Effect of the *pdeδ cdpk3* double mutant on average gliding speed of individual, morphologically mature ookinetes after 15–18 hour of culture. Statistical analysis was done by two-tailed t-test), p≤0.02 (A, C, D, F and G). For all graphs error bars show standard deviations in 3 biological replicates, * = significantly greater than *pdeδ*. Φ = significantly less than wild type. Ψ = significantly greater speed than *cdpk3* ookinetes.

In *P. falciparum* deletion of *pdeδ* results in a partial reduction of cGMP specific phosphodiesterase activity in late stage gametocytes, and in a concomitant increase in total cellular cGMP [30]. To examine whether the substrate specificity of *pdeδ* is conserved in *P. berghei*, we examined mixed asexual blood stages, gametocytes and ookinetes for cGMP specific PDE activity (Fig. 4A). As with *P. falciparum*, cGMP-PDE activity was similar in wild type and *pdeδ* schizonts but was reduced in *pdeδ* gametocytes. This reduction became less pronounced during ookinete formation, and once the ookinetes were fully mature cGMP-PDE levels again became indistinguishable between wild type and the *pdeδ* mutant. Deletion of *pdeδ* did not affect cAMP-PDE levels at any life cycle stage we studied (Fig. 4B). While confirming its substrate specificity in gametocytes, these data also show that *pdeδ* is not the dominant cGMP PDE in ookinete membrane extracts. We next measured total cellular cGMP content in purified ookinetes and found no marked differences between wild type, *pdeδ* and *gcβ* (Fig. 4C). One interpretation of these data is that PDEδ and GCβ do not contribute to the regulation of cGMP levels in ookinetes. Alternatively, with additional phosphodiesterases and probably guanylyl cyclase α still present in the mutants, global measurements in whole cell lysates may not reflect all physiologically relevant aspects cGMP levels adequately. To investigate the role of cGMP in ookinetes further we next asked if PKG (known to be activated by cGMP, but not cAMP) was required for expression of the *pdeδ* phenotype. Addition of 1 or 10 µM Cmpd 1 to ookinete cultures at 3 h, i.e. after gametogenesis and fertilisation had occurred, completely prevented dedifferentiation and resulted in morphologically normal ookinetes (Fig. 5A). This was consistent with the hypothesis that the dedifferentiation phenotype of the *pdeδ* mutant resulted from enhanced cGMP signalling leading to inappropriate activation of PKG. To test this hypothesis further, we asked if in a *gcβ pdeδ* double mutant the expected reduction in cGMP synthesis (and consequent PKG activation) would suppress the *pdeδ* phenotype. To obtain *gcβ pdeδ* parasites we mixed blood containing gametocytes of each single mutant and allowed cross-fertilisation to occur *in vitro*. Zygotes were then injected into the mosquito hemocoel and sporozoites transmitted back to mice by mosquito bite, where parasites were subjected to drug selection followed by dilution cloning. Three clones tested negative by Southern blot for intact *gcβ* and *pdeδ* genes and positive for the two gene targeting cassettes (Fig. 5B). In all *gcβ pdeδ* clones morphologically normal ookinetes formed (Fig. 5C), which had the reduced motility observed in the *gcβ* mutant (Fig. 5D). Since the *pdeδ* phenotype was not expressed in the double mutant, we conclude it requires an intact *gcβ* gene. That the *pdeδ* phenotype is thus epistatic with respect to *gcβ* supports a model in which both genes operate in the same cGMP dependent pathway upstream of PKG.

### Signalling through PKG compensates for loss of *cdpk3*

We next sought to establish the relationship between signalling through the calcium dependent CDPK3 kinase and the *gcβ*/*pdeδ*/*pkg* pathway. To study the genetic interactions between *pdeδ* and *cdpk3*, we selected double mutants among the progeny of a cross and genotyped these to verify disruption of both genes (Fig. 5E). Ookinetes from the *pdeδ cdpk3* clones underwent the characteristic dedifferentiation after forming (Fig. 5F). Thus unlike gc*β*, *cdpk3* was dispensable for expression of the *pdeδ* phenotype. We next compared the motility of the *cdpk3 pdeδ* mutant and of each single mutant to wild type (Fig. 5G). Measurements were taken 12–15 h after fertilisation, when motile ookinetes had already formed but had not yet dedifferentiated. As expected, the average gliding speed of *cdpk3* ookinetes at this time point was reduced if compared to wild type. In marked contrast, in the *cdpk3 pdeδ* double mutant the *cdpk3* phenotype was suppressed, revealing an unexpected genetic interaction between the cGMP and calcium dependent signalling pathways that regulate ookinete gliding.

## Discussion

Upon entering the mosquito midgut the sexual stages of malaria parasites must leave their protected intracellular niche and become exposed first to immune effector mechanisms of the host that remain active within the blood meal, and later to a toxic cocktail of digestive enzymes from the vector [40]. To escape this hostile environment and establish an infection in a mosquito, ookinetes rely critically on their ability to move through the blood meal and penetrate the peritrophic matrix and then cells of the midgut epithelium [8]. With a speed of only around 5 µm/min *in vivo* [8], *P. berghei* ookinetes are around 20 times slower than many other apicomplexan zoites [41]. It is possible that ookinetes do not need to cover large distances *in vivo* since the contraction of the midgut, which accompanies progression of digestion from the periphery towards the centre of the blood bolus, may contribute to bringing ookinetes close to the epithelium [20]. This may explain why, in spite of their gliding defect, *cdpk3* ookinetes accumulate in the periphery of the blood meal [16], in an area we think is the digestion zone, where they resist lysis for at least some time. The most critical function of gliding could thus be to enable ookinetes to cross the peritrophic matrix and penetrate the midgut epithelium.

By combining an *in vitro* gliding assay with pharmacological and experimental genetic approaches, the current study discovers interactions between signalling genes that affect ookinete motility and morphology and defines a cGMP signalling module consisting of the cGMP producing cyclase GCβ, the cGMP hydrolysing phosphodiesterase PDEδ, and the cGMP effector kinase PKG (see Fig. S4 for summary). *Plasmodium* and ciliate guanylyl cyclases have unusual structural and topological features when compared to mammalian cyclases [42], yet biochemical studies on recombinantly expressed *P. falciparum* C1 and C2 regions, which are predicted to combine to form the catalytic domain of GCβ, have demonstrated its guanylyl cyclase activity and a marked preference for GTP over ATP as substrate [28]. A strong motility defect described by Hirai *et al.* [27] for a *P. berghei gcβ* mutant, which we independently confirm in the present study, therefore implicated cGMP in regulating ookinete gliding, although it could not be ruled out that a critical role for the N-terminal ATPase-like domain of *gcβ* could on its own account for the phenotype.

Interestingly, it was a deletion mutant in *pdeδ*, a gene recently shown in *P. falciparum* to be important for cGMP hydrolysis at the gametocyte stage [30] that proved most informative. Deletion of *pdeδ* did not prevent the initial formation of ookinetes but resulted in their rounding up shortly after becoming motile. The change in cell shape was accompanied by a local breakdown of the inner membrane complex in some areas, whereas the apical complex remained ultrastructurally intact. Three lines of evidence suggest rounding up is a specific cellular dysregulation response, rather than a general precursor of parasite death: (1) *pdeδ* ookinetes do not go on to lyse in culture, (2) they have an active motor, and (3) they appear fully infectious when injected into the haemocoel, giving rise to normal salivary gland infections. However, forward gliding in *pdeδ* ookinetes is replaced with rotational movement. This is entirely consistent with ultrastructural evidence, which showed the subpellicular microtubules that determine orientation of the motor, running at a right angle to the anterior-posterior axis of the cell. Mosquito transmission of *pdeδ* parasites was strongly reduced, presumably because most ookinetes were unable to reach and penetrate the midgut epithelium in time before rounding up. It is tempting to speculate that increased cellular levels of cGMP resulting from deletion of *pdeδ* can prematurely initiate some of the cellular events that occur naturally once ookinetes have crossed the gut epithelium and reached the basal lamina. Ookinete-to-oocyst transformation involves the progressive breakdown of the IMC [43], a process we also observe in *pdeδ* ookinetes. However, natural transformation starts *in vitro* with a protrusion in the middle of the ookinete that always originates on the outer convex edge [44] and thus appears more clearly defined than the *pdeδ* phenotype. It is nevertheless conceivable that the partial loss of the IMC observed in *pdeδ* ookinetes could explain the change in cell shape, which in wild type is thought to be supported by a subpellicular network of filamentous proteins [45]. In fact, deletion of the *imc1b* gene, which encodes one of these proteins expressed in *P. berghei* ookinetes, results in a more rounded cell [46], however, the *imc1b* phenotype appears distinct from that of *pdeδ*. Additional work is required to investigate the relevance of the *pdeδ* phenotype for understanding ookinete shape and oocyst transformation.

We propose that PDEδ may not be directly involved in maintaining cell shape, but that the *pdeδ* phenotype is brought about by the inappropriate or untimely activation of signalling through cGMP. In support of this hypothesis, the *pdeδ* phenotype only appears from about 12 h in culture, which is shortly after the onset of ookinete motility, a process that depends on *gcβ*. Importantly the *pdeδ* phenotype is completely suppressed in *gcβ pdeδ* double mutants and by an inhibitor of PKG. Work in *P. falciparum* has identified the specific enzymatic activities of PDEδ and GCβ in cGMP metabolism [28]–[30], and we confirm here for *P. berghei* that deletion of PDEδ reduces cGMP specific PDE activity in gametocyte membrane extracts. Since the only factor to link both enzymes is therefore cGMP, it is the most logical interpretation of their genetic interaction that *pdeδ* and *gcβ* together exert critical control over cGMP levels in ookinetes. However, we also show that overall cGMP levels are not much affected in either *pdeδ* or *gcβ* mutant ookinetes, and other cGMP producing and degrading enzymes must therefore be present to control baseline cGMP levels in these cells. This is reminiscent of the situation in human platelets, where in the presence of multiple PDEs an isoform-selective inhibitor of PDE5, sildenafil, can impact platelet function not through increasing cGMP levels globally, but by acting locally on signalling complexes containing PDE5, PKG and its major substrate [47]. As a predicted transmembrane protein *Plasmodium* PDEδ is likely to function differently from human PDE5, which is soluble. However, the principle of compartmented cGMP signalling may well apply to highly polarised ookinetes, in which relevant changes in cGMP levels may need to be controlled in time and space and could be localised to a subcellular compartment, such as the submembrane space that accommodates the molecular motor. It is also possible that GCβ does not produce cGMP constitutively but only in actively moving ookinetes, which may be a small subset in a population of purified cells.

The ability of the PKG inhibitor, Cmpd 1, to block both ookinete gliding and expression of the *pdeδ* phenotype suggests that in both processes cGMP may act through PKG, which we show is expressed in ookinetes. The *pkg* gene is probably essential due to the likely role of cGMP signalling in asexual erythrocytic life cycle stages. We failed to delete or disrupt *pkg* but were able to insert a C-terminal GFP tag showing that the genome locus is in principle accessible to genetic modification. Ookinetes expressing the PKG-GFP protein instead of PKG had no phenotype, suggesting the tagged kinase was functional. PKG-GFP was evenly distributed throughout the cell and not enriched in the submembrane compartment that accommodates the motor. In contrast PDEδ and GCβ are predicted transmembrane proteins. GFP fusions of both were undetectable in ookinetes, suggesting both proteins are expressed at low levels. We were intrigued to find that in the *cdpk3 pdeδ* double knockout the *cdpk3* motility phenotype was suppressed, before the *pdeδ* phenotype appeared and prevailed. This would suggest that overstimulation of PKG could compensate for the loss of the calcium dependent CDPK3 kinase, which is also cytosolic [16],[20]. We can rule out that deletion of *cdpk3* has inadvertently interfered with the cGMP pathway, because complementation with an intact *cdpk3* gene expressed from an episome restored mosquito transmission [20]. Instead we propose that cGMP and calcium dependent pathways may converge at some point, for instance in a shared substrate for CDPK3 and PKG. Partial redundancy between both pathways might explain why motility is only incompletely blocked in *cdpk3* ookinetes.

Consistent with the ubiquitous expression of PKG in asexual erythrocytic, sexual and mosquito stages, signalling through cGMP is probably essential throughout the malaria life cycle. In a transgenic *P. falciparum* mutant expression of a Cmpd 1 resistant PKG allele rendered gametocyte activation by xanthurenic acid insensitive to Cmpd 1 [29], demonstrating at the same time the essential role of PKG early in gametocyte activation and confirming PKG as a critical target for Cmpd 1 in *Plasmodium*. The critical source for cGMP in gametocyte activation is most likely GCα, since mutants lacking GCβ form gametes as wild type in both *P. falciparum* [30] and in *P. berghei* ([27], and this study). While cGMP signals through the same effector kinase in different stages, second messenger production comes from stage specific pathways, presumably responding to distinct external or internal stimuli that remain to be identified. Hydrolysis of cGMP is also regulated in a stage specific manner, and is essential, probably to prevent over stimulation of PKG. Interestingly, PDEδ, which in *P. berghei* becomes essential only at the ookinete stage, has an earlier essential function in *P. falciparum*, where gene disruption leads to severely impaired gametogenesis, accompanied by a significant, although incomplete reduction in cGMP specific PDE activity in gametocytes [30]. Malaria parasites express four phosphodiesterases, at least two of which, PDEδ and PDEα [48] are selective for cGMP. Functional redundancy is thus likely, and subtle differences in expression timing of different PDEs, perhaps linked to the much extended maturation period of *P. falciparum* gametocytes, may account for the fact that in *P. berghei* PDEδ becomes essential later during sexual development. From our analysis of PDE activity and global cGMP measurements it emerges that multiple PDE enzymes are co-expressed in ookinetes that may serve different cellular functions. While these will be difficult to dissect biochemically, the focus on genetic interactions has allowed us to propose GCβ and PDEδ as a pair of functionally interacting cGMP regulator proteins important for gliding. Whether these interact physically in parasite membranes to form a signalling complex will be the subject of future work.

The essential role of cGMP mediated signal transduction in malaria transmission is of interest in view of recent efforts to target PKG and phosphodiesterases for antimalarial drug development. The prototype anticoccidial kinase inhibitor, Cmpd 1, is selective for parasite over host PKG and is effective in treating *Eimeria tenella* infections in chicken and acute *Toxoplasmosis* in mice [26],[36], although it is somewhat less effective against *P. berghei* [49]. Phosphodiesterases of humans are validated therapeutic targets and their highly divergent *Plasmodium* homologs appear to have distinct pharmacological properties [48],[50]. That PDE inhibitors compete with substrate concentrations 100 to 1000 times lower than ATP for protein kinases makes them intrinsically more likely to be effective [51], however, functional redundancy among *Plasmodium* PDEs [48] perhaps makes these less promising targets.

In principle, zoite gliding may be regulated at the level of apical secretion of transmembrane adhesins [1], assembly of the motor complex [25], actin polymerisation [52] and, through phosphorylation of the myosin light chain [24], at the level of the power stroke itself. In *T. gondii* for example, where convenient secretion assays are available, PKG is required for microneme secretion and thought to act downstream of a Ca^2+^ signal [26]. The current study has identified a critical function for a GCβ/PDEδ signalling module in regulating ookinete gliding and transmission of malaria, most likely by regulating the activity of PKG. How exactly this pathway interacts with calcium dependent signalling through CDPK3 and probably other calcium effector kinases remains to be resolved, and the molecular targets of PKG and CDPK3 need to be identified. Based on their diffuse cellular localisation alone, CDPK3 and PKG are likely to phosphorylate substrates distinct from those of CDPK1, which is anchored to the inner membrane of the plasmalemma [23],[24]. The availability of a quantitative ookinete gliding assay, combined with the relative genetic accessibility of this apicomplexan zoite stage now provides an opportunity to dissect the molecular pathways regulating zoite gliding.

## Materials and Methods

### Ethics statement

All animal work has passed an ethical review process and was approved by the United Kingdom Home Office. Work was carried out in accordance with the United Kingdom “Animals (Scientific Procedures) Act 1986” and in compliance with “European Directive 86/609/EEC” for the protection of animals used for experimental purposes.

### Bioinformatics

*P. berghei* cyclic nucleotide signalling genes were identified by searching the annotated 8× genome assembly (www.plasmodb.org) for relevant text words and protein domains. Sequence information and *P. berghei* annotated gene models were also obtained from the Wellcome Trust Sanger Institute website (www.sanger.ac.uk/Projects/P_berghei/). Orthologs in *P. falciparum* and *P. berghei* were identified as reciprocal best hits using the blastp algorithm. Where possible, *P. berghei* gene models were based upon experimentally confirmed models from *P. falciparum* orthologues. For some genes homology searches retrieved multiple sequences, which were predicted to be non-overlapping gene fragments from alignments with complete *P. falciparum* gene models. Where gene models were uncertain sequence was compared to gene models from other malaria genomes available on www.plasmodb.org, including *P. yoelii*, *P. knowlesi* and *P. vivax*. Tools used for identification of conserved domains and functional residues included Pfam (http://pfam.sanger.ac.uk/), SMART (http://smart.embl-heidelberg.de/), Interpro (www.ebi.ac.uk/InterProScan/) and Prosite (www.expasy.ch/prosite/).

### Parasite maintenance

All animal work was conducted under a license issued by the UK Home Office in accordance with national and international guidelines. The *P. berghei* ANKA wild type strain 2.34 and transgenic lines made in the same background were maintained in female phenyl hydrazine-treated Theiler's Original (TO) outbred mice as described previously [53] and infections monitored on Giemsa-stained blood films. Exflagellation was quantified 3 to 4 days post infection by adding 4 µl of blood from a superficial tail vein to 150 µl exflagellation medium (RPMI 1640 containing 25 mM HEPES, 4 mM sodium bicarbonate, 5% FCS, 100 µM xanthurenic acid, pH 7.3). Between 15 and 18 minutes after activation the number of exflagellating microgametocytes was counted in a haemocytometer and the red blood cell (RBC) count determined. The percentage of RBCs containing microgametocytes was assessed on Giemsa-stained smears and the number of exflagellations per 100 microgametocytes was then calculated. Ookinetes were produced *in vitro* by culturing gametocyte-infected mouse blood in ookinete medium (RPMI1640 containing 25 mM HEPES (Sigma), 10% FCS, 100 µM xanthurenic acid, pH 7.5) and conversion assays were performed by live staining of ookinetes and activated macrogametes with Cy3-conjugated 13.1 monoclonal antibody against P28. The conversion rate was determined as the number of banana shaped ookinetes as a percentage of the total number of Cy3-fluorescent cells. For time course experiments Giemsa- stained smears were prepared from the same culture every 3 h for 24 h. The percentage of mature ookinetes and transition stages was assessed for three independent cultures.

For transmission experiments batches of 50 female *A. stephensi*, strain SD500, mosquitoes were allowed to feed on infected TO mice three days after intraperitoneal injection of infected blood. Unfed mosquitoes were removed the day after, and mosquitoes were maintained on fructose at 19°C. Oocysts were counted on dissected midguts 11 days after feeding. Sporozoite numbers were determined on day 21 by homogenising dissected salivary glands and counting the released sporozoites. To determine sporozoites infectivity to mice, 21 d infected mosquitoes were allowed to feed on naïve C57BL/6 mice, which were then monitored daily for bloodstage parasites. For injection into the mosquito haemocoel, cultures were adjusted to 1.16×10^4^ ookinetes per µl, back-filled into borosilicate injector needles (Drummond) and 69 nl injected into the thorax of each of 100 adult female mosquitoes using a Nanoject II hand-held microinjector (Drummond).

### Ookinete motility assays

Ookinete cultures were added to an equal volume of Matrigel™ (BD) on ice, mixed thoroughly, dropped onto a slide, covered with a Vaseline-rimmed cover slip, and sealed with nail varnish. The Matrigel™ was then allowed to set at room temperature for at least 30 minutes. After identifying a field containing ookinetes, time-lapse videos (1 frame every 5 seconds, for 10 minutes) were taken of ookinetes using the differential interference contrast settings with a 63× objective lens on a Leica DMR fluorescence microscope and a Zeiss Axiocam HRc camera controlled by the Axiovision (Zeiss) software package. Speed of motility of individual ookinetes was measured by multiplying the number of body lengths moved by the length of the ookinete during the 10 minute video, divided by 10. Multiple independent slides and cultures were used for each parasite line. Video processing and annotations was carried out using the Axiovision or Axiovision LE (Zeiss) software.

### Production and genotyping of transgenic parasite lines

Targeting vectors for *gcβ* and *pdeδ* were constructed in plasmid pBS-DHFR, containing a *T. gondii dhfr/ts* expression cassette conveying resistance to pyrimethamine. Primers ol278 (CCCCCGGGCCCTATCGTTTACACTTTGTTTATGACGGTG) and ol279 (CCCCAAGCTTCAACAACACCATCAATATATTCGG) were used to amplify a 1 kb region of homology at the 5′ end of the *gcβ* locus, which was ligated between the *Apa*I and *Hin*dIII restriction sites, upstream of the *Tgdhfr/ts* marker. The construct was then linearised using an endogenous *Cla*I site, in preparation for transfection. For the *pdeδ* construct the 5′ flanking region was amplified with primers ol05 (GCGGGTACCGATATTGTACGCAAGTGGTAC) and ol06 (GCGATCGATGAATATCTCACTCATTCAAGC) which was ligated between the *Kpn*I and *Hin*dIII restriction sites upstream of the *Tgdhfr/ts* marker. The 3′ flanking region was amplified with the primers ol07 (GCGGAATTCCGGAATCCTAAATGACAAGTC) and ol08 (GCGACTAGTCCTCATCAGGTTTTTCCATAC), and ligated between the *Eco*RI and *Bam*HI restriction sites upstream of the *Tgdhfr/ts* marker. The final construct was excised by digestion with *Kpn*I and *Bam*HI for transfection of *P. berghei*, which was carried out as previously described [54]. After dilution cloning into naïve TO mice, integration of targeting vectors was confirmed with Southern blot analysis and diagnostic PCR using standard protocols.

### Crossing of transgenic lines

The *gcβ pdeδ* double KO line was produced by crossing single mutant clones *in vitro*. Blood containing similar numbers of gametocytes from *pdeδ* and *gcβ* mutants were added to ookinete medium and cultured for 24 h. The resultant ookinetes were then injected into mosquito haemocoel as described above. 21 days after injection mosquitoes were allowed to feed on naïve C57BL/6 mice to allow parasite transmission. After blood infections developed, parasites were put under pyrimethamine drug selection, and cloned by injecting limiting dilutions into naïve mice. Multiple clones were genotyped to identify double mutants. The *gcβ pdeδ* double KO clones were analysed in two separate Southern blots, using the digest/probe combinations from the *gcβ* and *pdeδ* Southern blots respectively. The *cdpk3 pdeδ* double KO lines were established in a similar manner, except that transmission of cross fertilised ookinetes was done through membrane feeding, rather than microinjection. Parasite clones were genotyped by Southern hybridisation with gene-specific probes, probes specific for the *tgdhfr/ts* resistance cassette, and by diagnostic PCR.

### Electron microscopy

Ookinetes, cultured for 24 h, were purified using paramagnetic beads coated with the monoclonal antibody 13.1 against the ookinete surface antigen P28, and resuspended in freshly prepared primary fixative containing 2% paraformaldehyde, 2% glutaraldehyde in 0.1 M sodium cacodylate buffer (pH 7.42) with added 0.1% magnesium chloride and 0.05% calcium chloride at 20°C for 2 hours. The suspension was centrifuged in a 1 ml tube at 3000 rpm for 5 minutes to form a stable pellet able to withstand further processing without disruption. The pellet was rinsed three times for 10 minutes each in sodium cacodylate buffer with added chlorides. Secondary fixation followed for 1 hour with 1% osmium tetroxide in sodium cacodylate buffer. The cells/beads were then rinsed 3 times in cacodylate buffer over 30 minutes and mordanted with 1% tannic acid for 30 minutes followed by a rinse with 1% sodium sulfate for 10 minutes. The samples were then dehydrated through an ethanol series 20%, 30% (staining en bloc with 2% uranyl acetate at this stage), 50%, 70%, and 90% for 20 minutes each, then 100% for 3×20 minutes. Ethanol was exchanged for propylene oxide (PO) for 2×15 minutes followed by 1∶1 PO to TAAB 812 resin kit for 1 hour and neat resin (with a few drops of PO) over night. The samples were embedded in a flat moulded tray with fresh resin and cured in an oven at 65°C for 24 h. 60 nm sections were cut on a Leica UCT ultramicrotome, contrasted with uranyl acetate and lead citrate and imaged on an FEI 120 kV Spirit Biotwin using an F415 Tietz CCD camera.

### Phosphodiesterase activity and intracellular cGMP measurements

Mixed asexual stages were purified from infected blood by lysing uninfected cells in red blood cell lysis buffer (0.15 M NH_4_Cl, 0.01 M KHCO_3_, 1 mM EDTA, pH 7.4), after removal of white blood cells by passing over a CF11 (Whatman) column. Gametocytes were purified on a nycodenz density gradient as previously described [55], and ookinetes were purified using paramagnetic beads coated with the monoclonal antibody 13.1 after 14 or 24 hours of culturing. Each parasite stage was then pelleted in aliquots of 10^7^ and 10^8^ cells, for cGMP and PDE measurements respectively, and then snap-frozen and stored at −80°C until use.

Phosphodiesterase activity in parasite membrane fractions was measured as described [30]. Parasites were lysed by subjection to freeze-thaw cycles in lysis buffer (20 mM HEPES, 250 mM sucrose, pH 7.0). Samples were then pelleted at 100,000 *g* and particulate fractions re-suspended in lysis buffer with EDTA-free protease inhibitors (Roche). PDE assays were carried out in triplicate on a 96-well plate in the presence of [^3^H]-labelled cGMP or [^3^H]-labelled cAMP (GE Healthcare) for 30 min at 37°C. Reactions were terminated by boiling for 1 min followed by a 3 min centrifugation at 900 *g*. 1 unit of alkaline phosphatase was then added and incubated for 30 min at 37°C. [^3^H]-labelled guanosine/adenosine was purified from the samples using ion exchange (Bio-Rad AG® 1×8 resin) and added to scintillation fluid (Optiphase Supermix, Wallac). Scintillation was measured using a Wallac 1450 Microbeta Liquid Scintillation Counter (Perkin Elmer) and PDE activity was expressed in pmol cNMP min^−1^ per 10^8^ cells. Intracellular cGMP concentrations of 24 h ookinete samples were determined in a radio immuno assay after freeze thawing samples in 0.5N perchloric acid and purifying cGMP as described [56].

## Supporting Information

Table S1 Putative cyclic nucleotide signalling genes in *P. falciparum* and *P. berghei*.
(0.16 MB TIF)

Figure S1 Guanyly cyclase and phosphodiesterase gene expression in *P. berghei*. Realtime PCR was carried out using cDNA from mixed asexual stages (mAS) purified from a gametocyte non-producer parasite strain (ANKA 2.33), and from purified gametocytes from strain ANKA 2.34. Relative expression levels of each gene were normalised to the expression levels of the elongation factor 1α gene and graphs calibrated to the lowest arbitrary value. The relative expression of (**A**) the two *P. berghei* guanylyl cyclase genes is shown as geometric means of triplicate biological samples and (**B**) expression of the four *P. berghei* phosphodiesterase genes is shown as the geometric mean of duplicate biological samples (+/−1 standard deviation).
(0.09 MB TIF)

Figure S2 Genotyping of *gcβ*, *pdeδ* and *cdpk3* mutant parasites. Schematic drawing illustrating the gene disruption strategy by single cross-over homologous recombination for *gcβ* (**A**) marked with positions of oligonucleotides used for diagnostic PCR (arrows), and probes and restriction sites used for Southern blot analysis. (**B**) Diagnostic PCR on genomic DNA showing integration of the *tgdhfr/ts* selection marker and absence of the intact *gcβ* gene in the clone 110.2. (**C**) Southern blot analysis of *Eco*RV-digested genomic DNA. A probe for the 5′ end of *gcβ* reveals a size shift consistent with the expected 3.2 kb fragment in wild type and a 5.5/5.6 kb doublet in *gcβ*. Schematic drawing illustrating the gene disruption strategy by double cross-over homologous recombination for *pdeδ* (**D**). (**E**) Diagnostic PCR on genomic DNA showing absence of wild type *pdeδ* in clone 97.1 and presence of a 1.2 kb band diagnostic of the 5′ integration event of the targeting vector. (**F**) Southern blot analysis of *Eco*RI-digested genomic DNA. A probe against the 5′ flanking region of *pdeδ* recognizes a 4.3 kb band in wild type parasites and reveals integration of the *pdeδ* replacement construct through a size shift to a 5.8 kb fragment in *pdeδ*. Schematic drawing illustrating the gene disruption strategy by double cross-over homologous recombination for *cdpk3* (**G**).
(0.31 MB TIF)

Figure S3 PKG-GFP is expressed and localised in the cytosol of different life cycle stages. The c-terminal GFP tag was added to the parasite PKG coding region through single crossover homologous recombination. (**A**) Confirmation by PCR on genomic DNA of correct integration of a tagging vector that fuses GFP in frame 3′ to the endogenous *pkg* by single cross-over homologous recombination. Positive control amplifies unrelated gDNA sequence. (**B**) Western blot analysis of purified ookinetes (Ook), mixed blood stages (mBS), schizonts (Sch) and gametocytes (Gam) from wild type (1) and PKG-GFP tagged parasites (2), probed with anti-GFP antibody (FL, Santa Cruz). In the upper panel the expected 125 kDa PKG-GFP protein is indicated. In the lower panel the TAT1 monoclonal antibody against tubulin was used as a loading control. (**C**) Fluorescence analysis of PKG-GFP expression in asexual blood stages (ABS), gametocytes (Gam; left panel activated male, right panel activated female) and ookinetes (Ook). Scale bar = 5 µm.
(2.18 MB TIF)

Figure S4 Summary of genetic and pharmacological analysis of cGMP signalling in the ookinete. Schematic summarizing the mutants/treatments used in this study along with their predicted effects on signalling through cGMP and mutant phenotypes.
(0.38 MB TIF)

Video S1 Wild type ookinete motility.
(3.30 MB MOV)

Video S2 *gcβ* ookinete motility.
(1.51 MB MOV)

Video S3 Motility of *pdeδ* ookinete with normal morphology.
(1.67 MB MOV)

Video S4 Motility of *pdeδ* ookinete with “stumpy” morphology.
(1.64 MB MOV)

Video S5 Motility of *pdeδ* ookinete with “round” morphology.
(1.26 MB MOV)
