# Supplementary figures and images for: A Cyclic GMP Signalling Module That Regulates Gliding Motility in a Malaria Parasite
Source: PLoS Pathog. 2009 Sep 25;5(9):e1000599. doi: 10.1371/journal.ppat.1000599 (PMC2742896; doi:10.1371/journal.ppat.1000599)

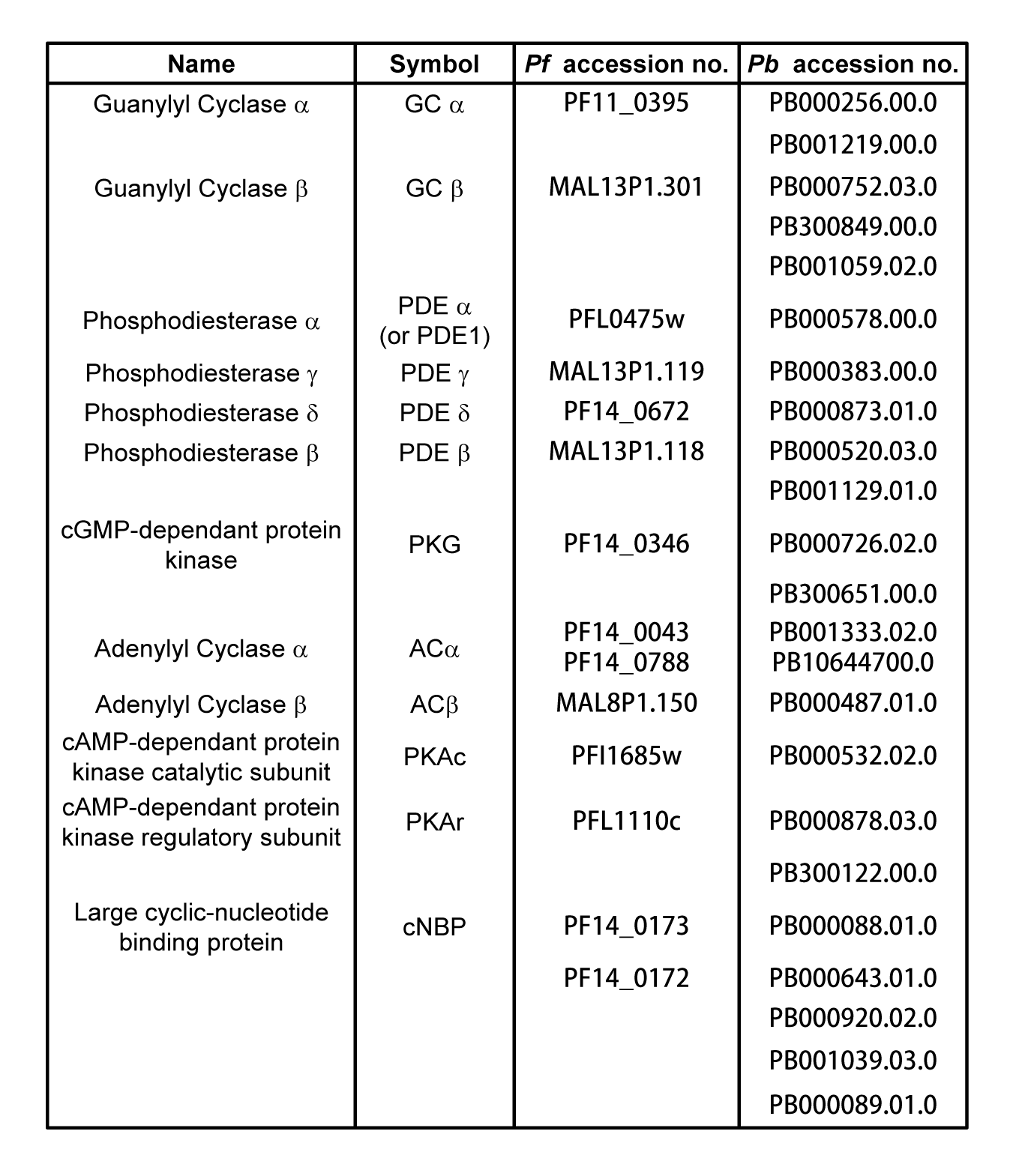

Supplement: Table S1 — Putative cyclic nucleotide signalling genes in P. falciparum and P. berghei. (0.16 MB TIF) [file ppat.1000599.s001.tif]

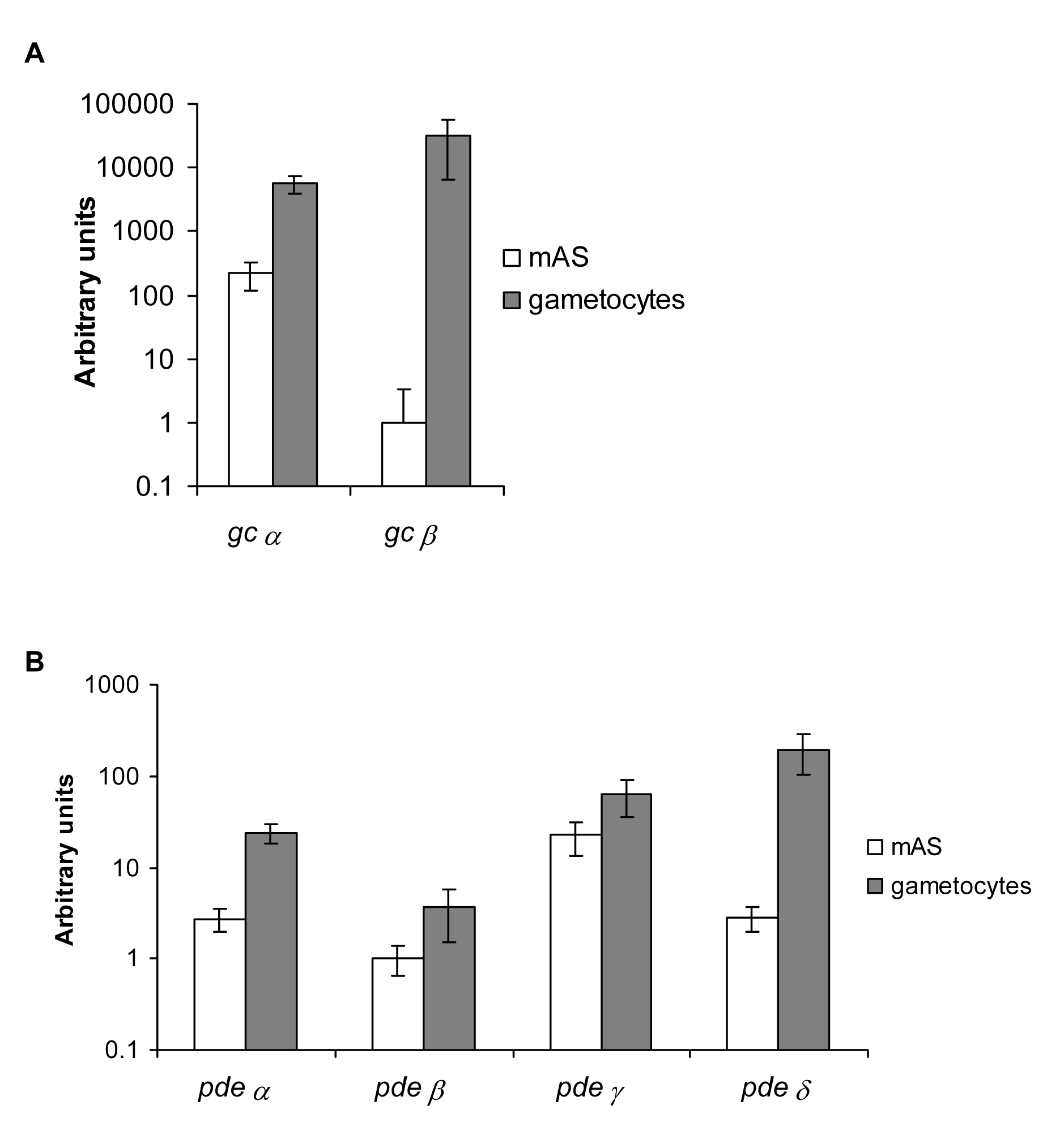

Supplement: Figure S1 — Guanyly cyclase and phosphodiesterase gene expression in P. berghei. Realtime PCR was carried out using cDNA from mixed asexual stages (mAS) purified from a gametocyte non-producer parasite strain (ANKA 2.33), and from purified gametocytes from strain ANKA 2.34. Relative expression levels of each gene were normalised to the expression levels of the elongation factor 1α gene and graphs calibrated to the lowest arbitrary value. The relative expression of (A) the two P. berghei guanylyl cyclase genes is shown as geometric means of triplicate biological samples and (B) expression of the four P. berghei phosphodiesterase genes is shown as the geometric mean of duplicate biological samples (+/−1 standard deviation). (0.09 MB TIF) [file ppat.1000599.s002.tif]

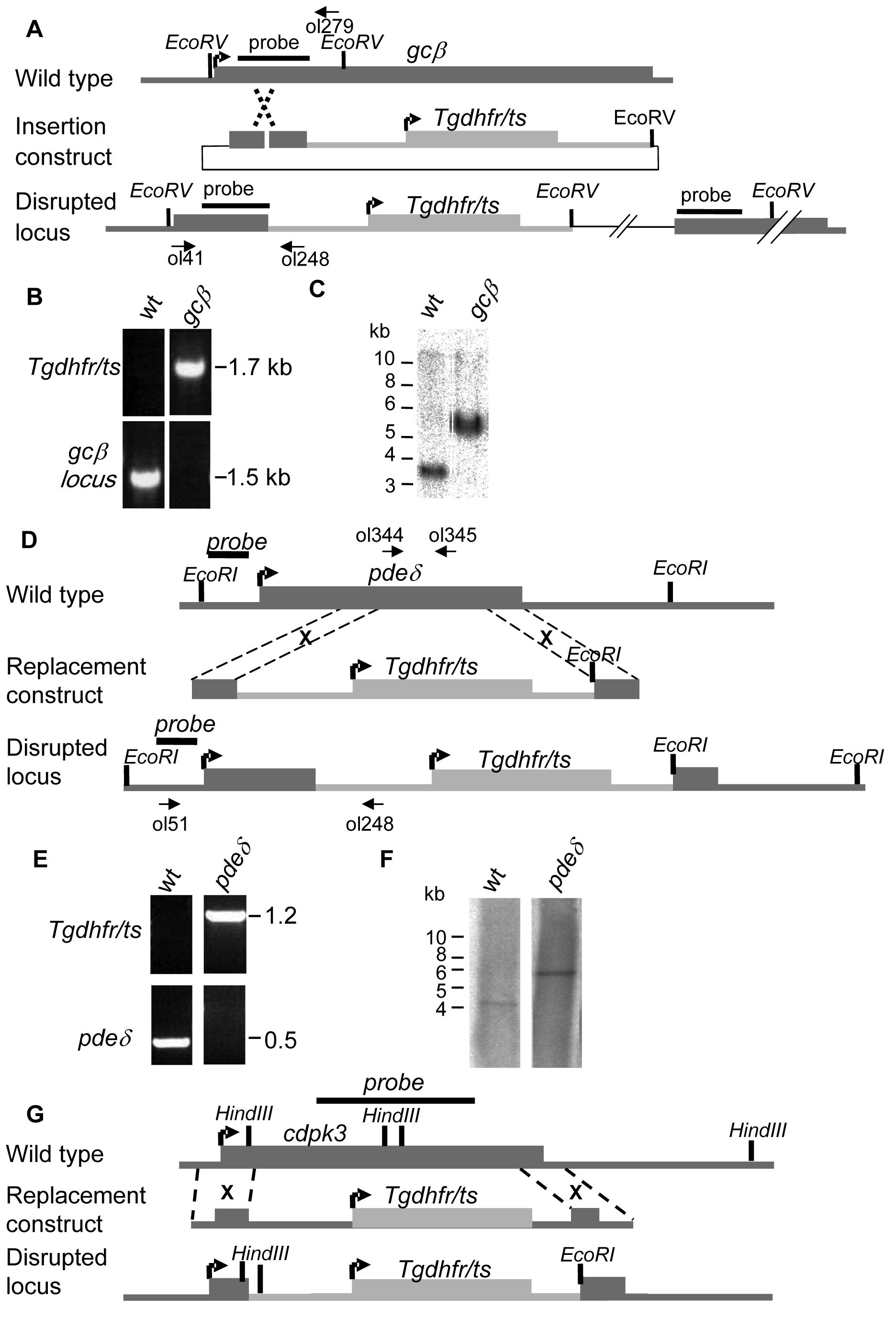

Supplement: Figure S2 — Genotyping of gcβ, pdeδ and cdpk3 mutant parasites. Schematic drawing illustrating the gene disruption strategy by single cross-over homologous recombination for gcβ (A) marked with positions of oligonucleotides used for diagnostic PCR (arrows), and probes and restriction sites used for Southern blot analysis. (B) Diagnostic PCR on genomic DNA showing integration of the tgdhfr/ts selection marker and absence of the intact gcβ gene in the clone 110.2. (C) Southern blot analysis of EcoRV-digested genomic DNA. A probe for the 5′ end of gcβ reveals a size shift consistent with the expected 3.2 kb fragment in wild type and a 5.5/5.6 kb doublet in gcβ. Schematic drawing illustrating the gene disruption strategy by double cross-over homologous recombination for pdeδ (D). (E) Diagnostic PCR on genomic DNA showing absence of wild type pdeδ in clone 97.1 and presence of a 1.2 kb band diagnostic of the 5′ integration event of the targeting vector. (F) Southern blot analysis of EcoRI-digested genomic DNA. A probe against the 5′ flanking region of pdeδ recognizes a 4.3 kb band in wild type parasites and reveals integration of the pdeδ replacement construct through a size shift to a 5.8 kb fragment in pdeδ. Schematic drawing illustrating the gene disruption strategy by double cross-over homologous recombination for cdpk3 (G). (0.31 MB TIF) [file ppat.1000599.s003.tif]

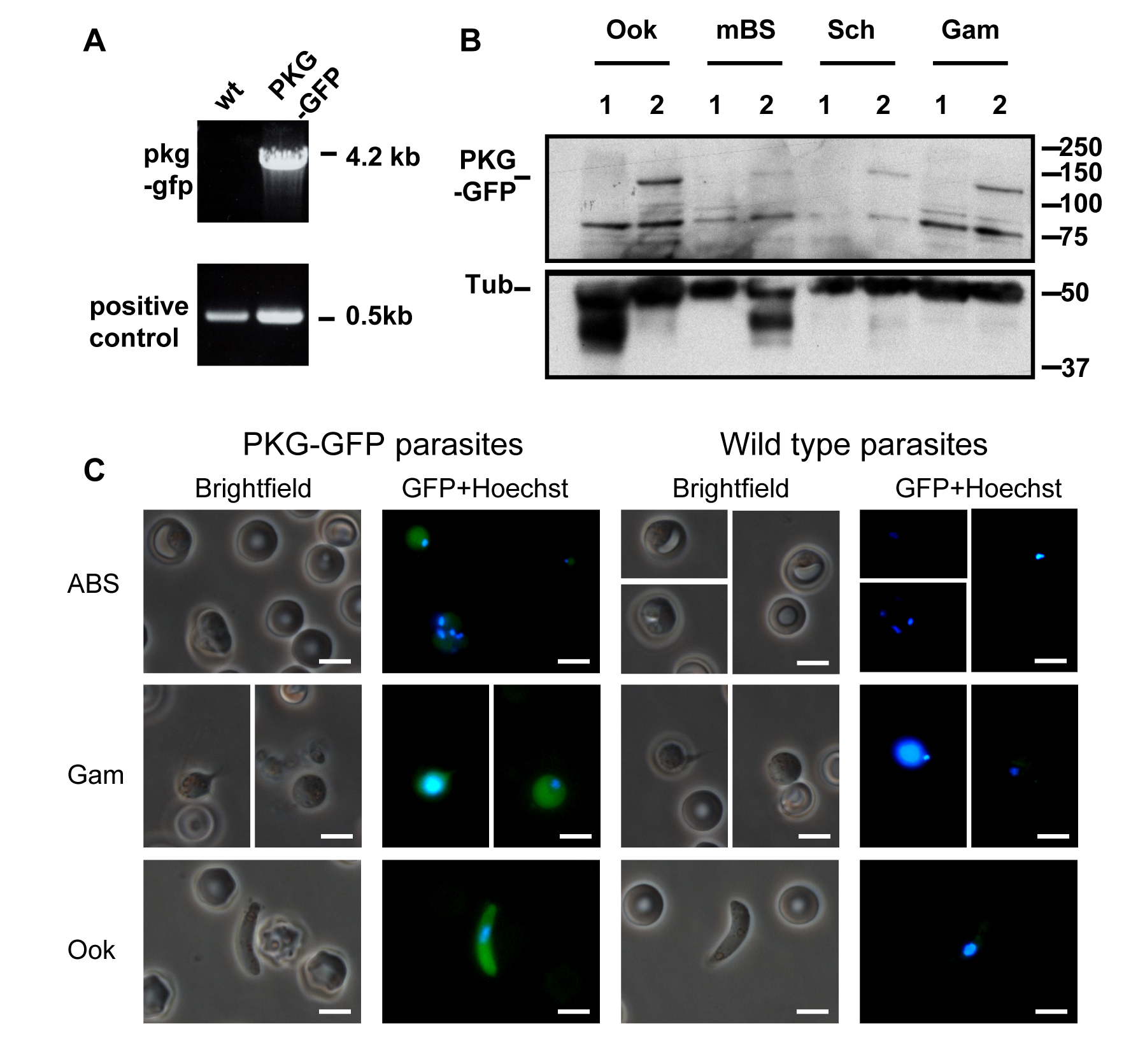

Supplement: Figure S3 — PKG-GFP is expressed and localised in the cytosol of different life cycle stages. The c-terminal GFP tag was added to the parasite PKG coding region through single crossover homologous recombination. (A) Confirmation by PCR on genomic DNA of correct integration of a tagging vector that fuses GFP in frame 3′ to the endogenous pkg by single cross-over homologous recombination. Positive control amplifies unrelated gDNA sequence. (B) Western blot analysis of purified ookinetes (Ook), mixed blood stages (mBS), schizonts (Sch) and gametocytes (Gam) from wild type (1) and PKG-GFP tagged parasites (2), probed with anti-GFP antibody (FL, Santa Cruz). In the upper panel the expected 125 kDa PKG-GFP protein is indicated. In the lower panel the TAT1 monoclonal antibody against tubulin was used as a loading control. (C) Fluorescence analysis of PKG-GFP expression in asexual blood stages (ABS), gametocytes (Gam; left panel activated male, right panel activated female) and ookinetes (Ook). Scale bar = 5 µm. (2.18 MB TIF) [file ppat.1000599.s004.tif]

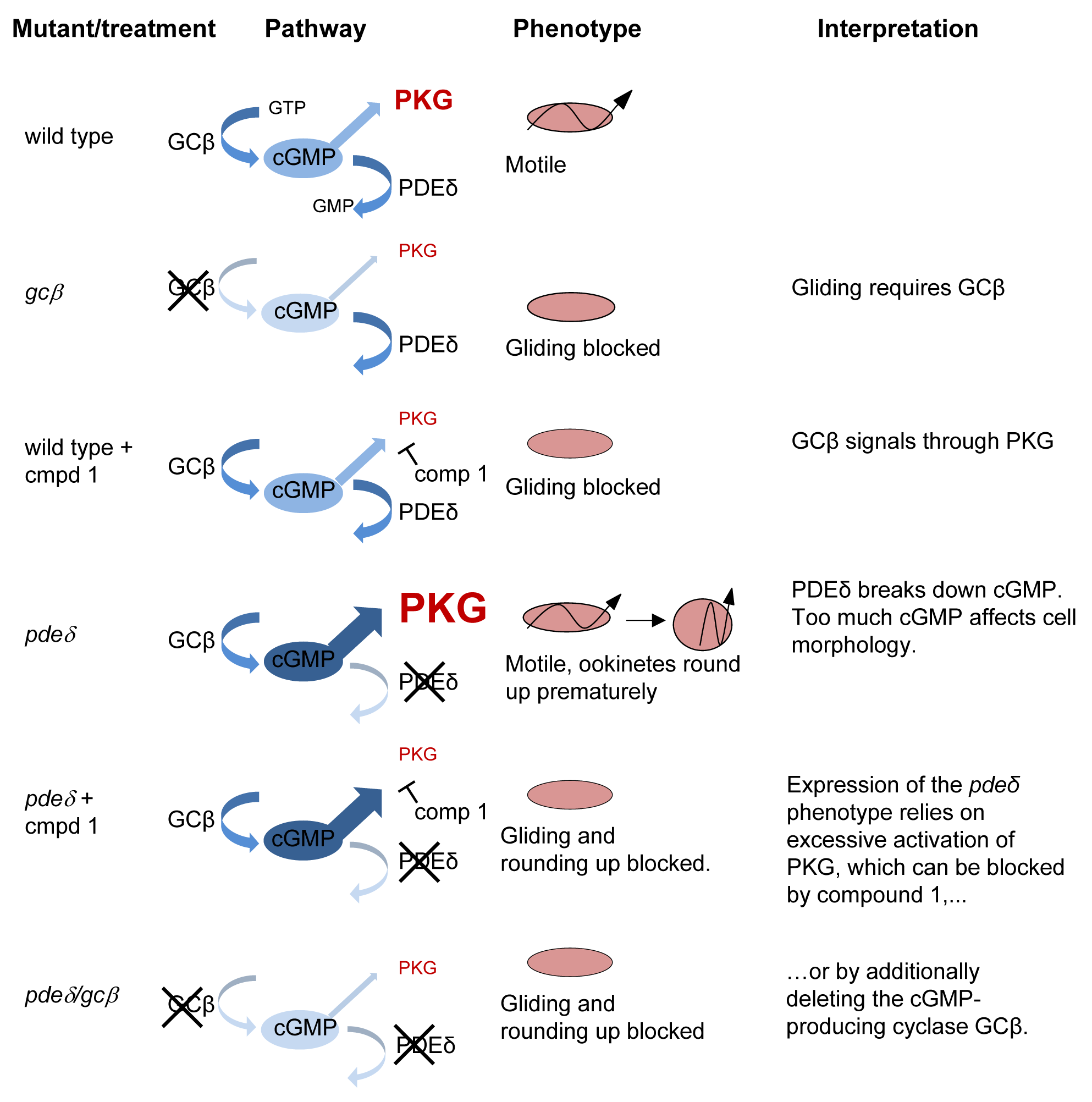

Supplement: Figure S4 — Summary of genetic and pharmacological analysis of cGMP signalling in the ookinete. Schematic summarizing the mutants/treatments used in this study along with their predicted effects on signalling through cGMP and mutant phenotypes. (0.38 MB TIF) [file ppat.1000599.s005.tif]
